# Inward currents induced by ischemia in rat spinal cord dorsal horn neurons

**DOI:** 10.1186/1744-8069-3-10

**Published:** 2007-04-25

**Authors:** Meng Chen, Yuan-Xiang Tao, Jianguo G Gu

**Affiliations:** 1 Department of Oral and Maxillofacial Surgery, McKnight Brain Institute and College of Dentistry, University of Florida, Gainesville, Florida, 32610, USA; 2 Department of Anesthesiology and Critical Care Medicine, Johns Hopkins University School of Medicine, 355 Ross, 720 Rutland Ave., Baltimore, Maryland 21205, USA

## Abstract

Hypoxia and ischemia occur in the spinal cord when blood vessels of the spinal cord are compressed under pathological conditions such as spinal stenosis, tumors, and traumatic spinal injury. Here by using spinal cord slice preparations and patch-clamp recordings we investigated the influence of an ischemia-simulating medium on dorsal horn neurons in deep lamina, a region that plays a significant role in sensory hypersensitivity and pathological pain. We found that the ischemia-simulating medium induced large inward currents in dorsal horn neurons recorded. The onset of the ischemia-induced inward currents was age-dependent, being onset earlier in older animals. Increases of sensory input by the stimulation of afferent fibers with electrical impulses or by capsaicin significantly speeded up the onset of the ischemia-induced inward currents. The ischemia-induced inward currents were abolished by the glutamate receptor antagonists CNQX (20 μM) and APV (50 μM). The ischemia-induced inward currents were also substantially inhibited by the glutamate transporter inhibitor TBOA (100 μM). Our results suggest that ischemia caused reversal operation of glutamate transporters, leading to the release of glutamate via glutamate transporters and the subsequent activation of glutamate receptors in the spinal dorsal horn neurons.

## Background

Glutamate is the principle neurotransmitter that mediates sensory transmission in the spinal cord dorsal horn. Under physiological conditions, glutamate is released synaptically by primary afferent fibers, descending terminals from supraspinal regions, and excitatory interneurons in the spinal cord dorsal horn [1]. The synaptically released glutamate is rapidly taken up through glutamate transporters located at presynaptic terminals, postsynaptic cells, and on the surrounding glia cells [2-5]. These transporters keep extracellular glutamate at low levels to ensure high fidelity sensory transmission, to limit nonspecific neuronal excitation and hyperactivity, and to prevent excitatory toxicity [3,6].

Increased glutamate concentrations in extracellular spaces can occur as a consequence of CNS tissue injury, which in turn can produce neuronal hyperactivity and secondary neuronal tissue damage due to excitatory toxicity [7]. It has been shown that extracellular glutamate levels increased significantly in the brain following ischemic and hypoxic injury [8,9]. In the spinal cord, ischemia and hypoxia can occur under a number of pathological conditions including traumatic spinal cord injury, tumors within the spinal cord, spinal stenosis, cardiac arrest, massive hemorrhagic shock, and surgical procedures [10-14]. These conditions often cause spinal blood vessel compression, resulting in spinal cord ischemia and hypoxia. Similar to the brain, spinal cord ischemia and hypoxia also can result in the increases of extracellular glutamate levels to cause neuronal excitatory toxicity in the spinal cord. When these pathological processes occur in the dorsal horn of the spinal cord, sensory functions may be significantly altered to result in pathological pain states.

Leak of glutamate from damaged cells and release of glutamate from synaptic sites were thought to contribute to the elevation of extracellular glutamate concentrations under pathological conditions. However, studies have suggested that a change of glutamate transporter function plays a critical role in the sustained elevation of extracellular glutamate levels during ischemia and hypoxia [9,15]. Under physiological conditions, glutamate transporters co-transport one glutamate molecule and 3 Na^+ ^ions into the cell to maintain the concentration gradient of micromolar extracellular glutamate against millimolar intracellular glutamate [16,17]. This active transport function is supported by the transmembrane ion gradients established by Na^+^-K^+ ^ATPase [16,17]. Under pathological conditions, for example, during brain ischemia and hypoxia, ATP is depleted and Na^+^-K^+ ^ATPase function is impaired. This subsequently results in the loss of transmembrane ion gradients and thereby reducing the driving force for the active uptake of glutamate from extracelular glutamate [15]. In fact, studies using brain tissues suggested that the depletion of intracellular energy not only compromises glutamate uptake, but also can result in glutamate release through glutamate transporter system due to the reversal operation of the glutamate transporters [9].

In the present study, we tested the hypothesis that ischemic condition results in the reversal operation of glutamate transport system to cause glutamate release and subsequent excitation of sensory neurons in the spinal cord dorsal horn. The study may have implications in pathological pain states associated with ischemic and hypoxic conditions in the spinal cord dorsal horn [5].

## Results

**Figure 1:**
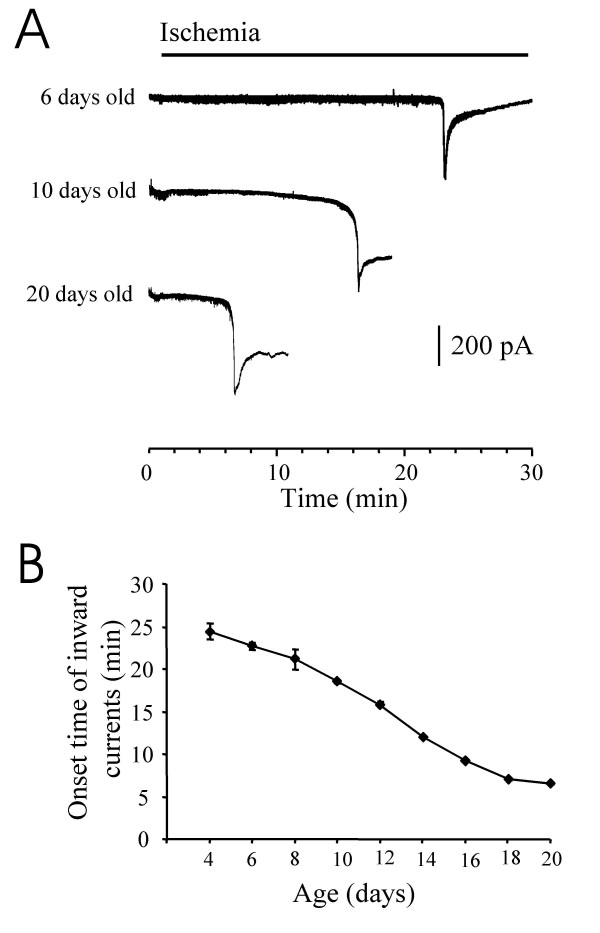
**Ischemia-induced inward currents in spinal cord dorsal horn neurons of rats at different ages**. **A)**. Three sample traces show inward currents induced by ischemia in dorsal horn neurons from rats at the age of 6 days old (top trace), 10 days old (middle trace), and 20 days old (bottom trace). The horizontal bar above the traces indicates the time period when ischemia bath solution was perfused to the spinal cord slice preparations. **B)**. The relationship between ages of rats and onset time of ischemia-induced inward currents. Numbers above the curve indicates the number of animals. All recordings were made from lamina V dorsal horn neurons.

The ischemic condition was produced by the bath application of a modified Kreb's solution that did not contain glucose and was bubbled with N_2 _gas to deoxygenate the solution. The bath solution also contained 1 mM sodium cyanide to block glycolysis and oxidative phosphorylation [9]. When this ischemia-simulating medium [18,19] was perfused to the spinal cord slice preparations, we recorded large inward currents (ischemia-induced inward currents) from lamina V neurons of the spinal cord dorsal horn (Fig 1A). The onset time of the ischemia-induced inward currents showed large variations. When animals at the age of 6 days old were used, the onset of the ischemia-induced inward currents was at 22 ± 1 min (n = 6, Figure 1A&1B). The ischemia-induced inward currents reached peak levels rapidly and then gradually decayed to baseline levels in recordings when the spinal cord slices were prepared from these young animals (Figure 1A). The onset time of the ischemia-induced inward current became shorter when older animals were used. For animals at the age of 10 days old, the onset of the ischemia-induced inward currents was at 18.5 ± 0.5 min (n = 6). When animals at the age of 20 days old were used, the onset time was 8 ± 0.4 min (n = 6), 3 times shorter than the onset time for animals at the age of 6 days old (Figure 1). We observed that, in older animals, the ischemia-induced inward currents usually did not return to baseline and membrane seals on patch-clamp electrodes were eventually lost (Figure 1A). The age-dependence in the onset of the ischemia-induced inward currents was observed in the range from 4 days to 20 days old (Figure 1B); animals over 20 days old were not examined in this study. The peak amplitude of the ischemia-induced inward currents, on the other hand, was not found to be age-dependence. Overall, the peak amplitude was 720 ± 38 pA (n = 43)

**Figure 2:**
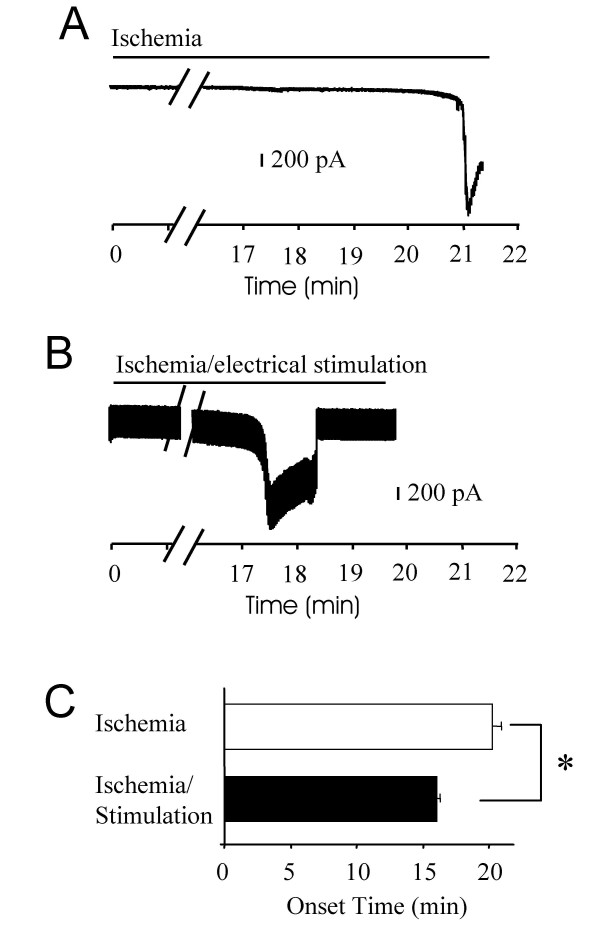
**Shortening of the onset time of ischemia-induced inward currents by capsaicin stimulation**. **A)**. A sample trace shows ischemia-induced inward current in a lamina V neuron of a rat at the age of 9 days old. **B)**. A sample trace shows ischemia-induced inward currents in a lamina V neuron of another rat at the age of 9 days old. In this experiment, focal stimulation (100 μA, 100 Hz) was applied to dorsal root entry zone while ischemic bath solution was applied to the spinal cord slice preparation. Note that in this sample trace, stimulation artifacts were superimposed on the inward currents. **C)**. Pooled results from experiments represented in **A **(open bar, n = 6) and **B **(solid bar, n = 4). All animals used were at the age of 9 days old.

We tested whether sensory input affects the ischemia-induced inward currents. Sensory input into spinal cord dorsal horn was elicited by electrical stimulation of primary afferent fibers at the intensity of 100 μA and frequency of 100 Hz. In this set of experiments, all the spinal cord slices were prepared from animals at the age of 9 days old. When primary afferent fibers were not stimulated electrically, the onset of the ischemia-induced inward currents was at 20 ± 0.6 min (n = 6) following the application of ischemic solution (Figure 2A&2C). On the other hand, the onset time was 16 ± 0.3 (n = 4) min when primary afferent fibers were repeatedly stimulated at the frequency of 100 Hz (Figure 2B&2C), significantly shorter than the onset time when primary afferent fibers were not stimulated (P < 0.05, Figure 2C). There was no significant differences in the peak current amplitude between the experiments without electrical stimulation (1400 ± 350 pA, n = 6) and the experiments with electrical stimulation (1359 ± 345 pA, n = 4).

**Figure 3:**
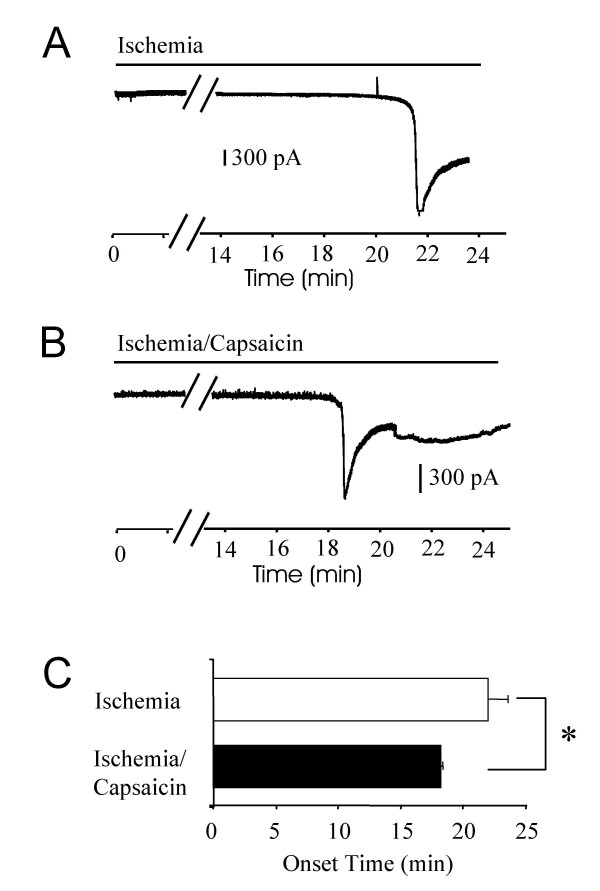
**Shortening of the onset time of ischemia-induced inward currents by electrical stimulation**. **A)**. A sample trace shows ischemia-induced inward current in a lamina V neuron of a rat at the age of 9 days old. **B)**. A sample trace shows ischemia-induced inward currents in a lamina V neuron of another rat at the age of 9 days old. In this experiment, capsaicin (2 μM) was applied in ischemic bath solution to the spinal cord slice preparation. **C)**. Pooled results from experiments represented in **A **(open bar, n = 6) and **B **(solid bar, n = 4). All animals used were at the age of 9 days old.

We tested whether stimulation of TRPV1-expressing nociceptive afferent fibers with capsaicin also affect ischemia-induced inward currents. In this set of experiments, all the spinal cord slices were prepared from animals at the age of 7 days. In experiments when capsaicin was not applied, ischemia-induced inward currents had onset time of 22 ± 1.8 min (n = 6) following the application of the ischemic Kreb's solution (Figure 3A&3C). When capsaicin (2 μM) was included in the ischemic solution, the onset time of the inward currents was 18 ± 0.2 min (n = 4; Fig 3B&3C), significantly shorter than the onset time of the experiments when capsaicin was absent (P < 0.05, Figure 3C). There was no significant differences in the peak current amplitude between the experiments without capsaicin stimulation (805 ± 395 pA, n = 6) and the experiments with capsaicin stimulation (765 ± 120 pA, n = 4).

**Figure 4:**
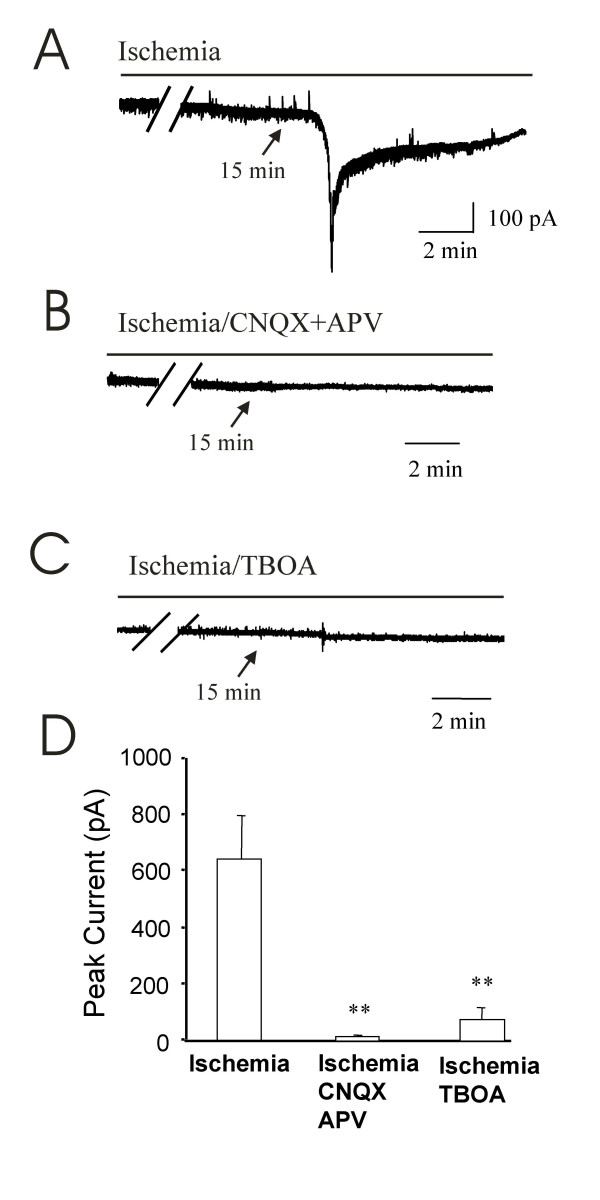
**Blocking of ischemia-induced inward currents by ionotropic glutamate receptor antagonists and glutamate transporter inhibitors**. **A)**. A sample trace shows ischemia-induced inward currents from a lamina V neuron of a rat at the age of 10 days old. **B)**. A sample trace shows the block of ischemia-induced inward currents by the glutamate receptor antagonists CNQX (20 μM) and APV (50 μM). The recording was made from a rat different from **A**. **C)**. Inhibition of ischemia-induced inward currents by the glutamate transporter inhibitor TBOA (100 μM). The recording was made from a rat different from A and B. **D)**. Summary of ischemia-induced inward currents in the absence (first bar, n = 4), in presence of CNQX and APV (n = 6), or in the presence of TBOA (n = 9). All recordings were made from lamina V neurons of the rats at the age of 10 days old.

We explored mechanisms by which ischemia induced inward currents. In this set of experiments, all animals used were at the age of 10 days old. The ischemia-induced inward currents had an onset time of ~17 min (Figure 4A, also see Figure 1B) with peak currents of 646 ± 150 pA (n = 4). In the presence of glutamate receptor antagonists CNQX (20 μM, for non-NMDA receptors) and APV (50 μM, for NMDA receptors), ischemia-induced inward currents were almost completely abolished (16 ± 2 pA, n = 6, p > 0.05; Figure 4B&4D). In the presence of DL-threo-β-benzyloxyaspartate (TBOA, 100 μM), a potent glutamate transporter inhibitor, the ischemia-induced inward currents were also largely inhibited (Figure 4C&4D). The ischemia-induced inward currents were 77 ± 36 pA (n = 9) in the presence of TBOA, only 12% of the ischemia-induced inward currents in those experiments when TBOA was absent (Figure 4D).

**Figure 5:**
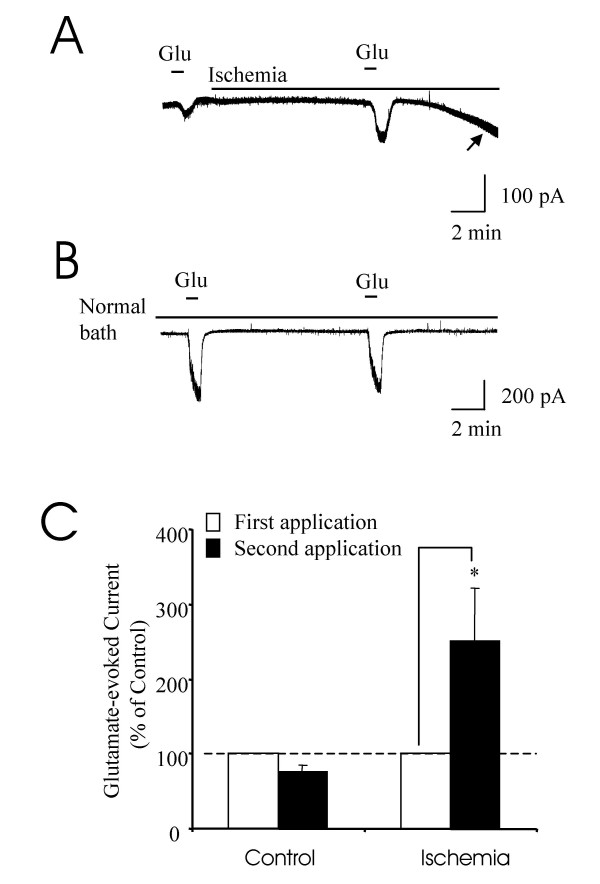
**Inward currents evoked by exogenous glutamate under ischemia conditions**. **A)**. A sample trace shows inward currents elicited by two applications of exogenous glutamate. The first application was made in normal bath solution and the second application was made 10 min following the perfusion of ischemia bath solution. An arrow indicates the start of the ischemia-induced inward currents. **B)**. A sample trace shows inward currents elicited by two applications of exogenous glutamate in normal bath solution. **C)**. Summary of the changes of glutamate-evoked currents in normal bath solution (first set of bars) and in ischemic bath solution (second set of bars). The current amplitudes were normalized. Open bars represent the currents evoked by the first application recordings of glutamate (n = 6); black bars represent the currents evoked by the second applications of glutamate (n = 6).

We determined whether ischemic conditions have a significant effect on inward currents evoked by exogenously applied glutamate. In this set of experiments, glutamate (500 μM, 1 min) was first applied for 1 min under the normal Kreb's bath condition. We then switched the normal bath solution to ischemic bath solution. Following a 10-min perfusion of ischemic bath solution, a second application of glutamate (500 μM, 1 min) was tested. This application was made prior to the onset of the ischemia-induced inward currents (Figure 5A). We found that the inward current evoked by the second glutamate application was 250 ± 65% of the inward current induced by the first glutamate application (p < 0.05, n = 6) (Figure 5A&5C). In contrast, when tissues were maintained at normal Kreb's bath solution, the inward current elicited by the second application of glutamate was usually slightly smaller than the current elicited by the first glutamate application (78 ± 8%, p > 0.05, n = 6, Figure 5B&5C).

## Discussion

In this study, we show that ischemia induced significant inward currents in dorsal horn neurons of the spinal cord and that the inward currents could be completely blocked by ionotropic glutamate receptor inhibitors as well as by glutamate transporter inhibitors. This observation is consistent with a previous study using brain slice preparations showing that glutamate transporter inhibitors blocked ischemia-induced inward currents [9]. These findings suggest that ischemia may cause reversal operation of glutamate transporters, leading to the release of glutamate via glutamate transporters and the subsequent activation of glutamate receptors in the neurons of the spinal dorsal horn and brain. Our study, however, cannot exclude other mechanisms that may contribute to ischemia-induced membrane depolarization as shown in hippocampal neurons [18,19].

We observed that the onset of ischemia-induced inward currents took many minutes. This delay in the onset suggests that intracellular energy depletion is a slow process under our experimental conditions and transporter reversal does not occur before intracellular energy is substantially depleted. Interestingly, we have found that the onset times of ischemia-induced currents are age-dependent, being shorter in older animals and longer in younger ones. One explanation for the age-dependence is that the rates of intracellular energy depletion may be different between younger and older animals under ischemic conditions. The onset times of ischemia-induced currents were shortened when primary afferent fibers were stimulated electrically or with the noxious stimulant capsaicin. This is mort likely due to fast energy depletion when primary afferent fibers were stimulated. In addition to the demonstration of ischemia-induced inward currents, we have also showed that inward currents evoked by exogenous glutamate were significantly larger under ischemia condition than under normal condition. This is probably because the actual concentrations of exogenous glutamate that reached the recorded neurons were higher under ischemia condition than under normal condition. This result suggest that prior to the reversal operation of glutamate transporters, glutamate uptake is severely compromised under ischemia condition. Glutamate transporters are expressed on both neuronal and glia cells in the spinal cord dorsal horn [4,5]. To date, five subtypes of glutamate transporters have been cloned: GLAST (EAAT1), GLT-1 (EAAT2), EAAC-1 (EAAT3), EAAT4 and EAAT5 [6]. GLAST, and GLT-1 are predominantly localized in astrocytes [2,20,21], while EAAC-1, EAAT4, and EAAT5 appears to be mostly neuronal [22-25]. While both neuronal and glial glutamate transporters actively participate in the uptake of extracellular glutamate [26,27]. Glutamate-induced excitatory toxicity under ischemia conditions appears to be mainly due to the impairment of glial glutamate transporters [3,28,29].

Previous studies have demonstrated that TBOA blocks glutamate uptake under physiological conditions [30]. We showed that ischemia-induced glutamate release was inhibited by glutamate transporter inhibitor TBOA in the present study. These results together suggest that TBOA bi-directionally blocks glutamate transporters. Effects of TBOA on sensory behaviors have previously been studied in both normal animals and animals with pathological pain conditions. In normal animals, intrathecal injection of TBOA was shown to induce nociceptive behaviors, such as licking, shaking, and caudally directed biting [31]. These effects were thought due to the block of glutamate uptake by TBOA, which subsequently results in the elevation of extracellular glutamate levels to cause hyperactivity in the spinal cord dorsal horn neurons [31]. Interestingly, under pathological pain conditions, glutamate transporter inhibitors were found to produce anti-nociceptive effects. For example, glutamate transporter inhibitors were shown to attenuate the induction of allodynia induced by PGE2, PGF2a, and NMDA [32], to reduced formalin-induced nociceptive responses, and to attenuate Complete Freund's adjuvant (CFA)-evoked thermal hyperalgesia [5,33]. In addition, transient knockdown of spinal GLT-1 led to significant reduction of nociceptive behavior in the formalin model [5,33]. It has been proposed that pathological pain conditions may cause a depletion of intracellular energy in the spinal cord dorsal horn, which subsequently reverses glutamate transporters to release glutamate and to produce hyperactivity in the spinal cord dorsal horn neurons [5]. Therefore, by blocking glutamate release from its transporters, glutamate transporter inhibitors may execute an anti-nociceptive effect under pathological conditions [5]. The present *in vitro *study supports the rationales for the use of glutamate transporter inhibitors.

## Methods

Principles of laboratory animal care (NIH publication No. 86-23, revised 1985) were followed in all the experiments described in the present study. Spinal cord slice preparations and patch-clamp recordings were described in details in our previous studies [34]. In brief, Sprague Dawley rats at the postnatal age of 4–21 days were used. Transverse spinal cord slices were prepared from lumbar enlargement of the spinal cords. The thickness of each slice was 400 μm. The spinal cord slices were maintained in a basket submerged in ~200 ml Krebs solution at 24°C. The Krebs solution contained (in mM): NaCl 117, KCl 3.6, CaCl_2 _2.5, MgCl_2 _1.2, NaH_2_PO_4 _1.2, NaHCO_3 _25 and glucose 11; the solution was saturated with 95 % O_2 _and 5% CO_2 _and had pH of 7.4. In each experiment, a spinal cord slice was transferred to a recording chamber and placed on the stage of an upright IR-DIC microscope. The slice was perfused at ~10 ml/min with the Krebs solution and all experiments were performed at room temperature (24°C). To induce ischemia, the slices were perfused by a ischemic bath solution, which is modified Kreb's solution that had no glucose (replaced with 7 mM sucrose) and contained 1 mM sodium cyanide [9], and the bath solution was deoxygenated by continuously bubbling with 100% nitrogen.

Individual neurons were identified under an IR-DIC microscope with a 40× water immersion objective. Whole cell patch-clamp recordings were made in deep lamina (lamina V) neurons with electrodes that were filled with an internal solution contained (mM): Cs_2_SO_4 _110, TEA-Cl 5, CaCl_2 _0.5, MgCl_2 _2, EGTA 5, HEPES 5, pH 7.3. The resistance of electrodes was ~5 MΩ when filled with the internal solution. The access resistance was below 30 MΩ and was not compensated. Signals was amplified and filtered at 2 kHz (Axopatch 200B) and sampled at 5 kHz using pCLAMP 7.0 (Axon Instruments). In all recordings, neurons were voltage-clamped at -30 mV. In some experiments, primary afferent fibers were stimulated by focal electrical stimulation at the dorsal root entry zone or by chemical stimulation with capsaicin. For the focal electrical stimulation, the stimulation intensity was 100 μA and the stimulation frequency was 100 Hz. For chemical stimulation, capsaicin at the concentration of 2 μM was bath applied to stimulate capsaicin-sensitive nociceptive afferent fibers. Pharmacological tests, including the tests of CNQX (20 μM), APV (50 μM), and TBOA (100 μM) were performed by applications of these compound through bath solution. Unless otherwise indicated, all recordings were performed in the presence of 20 μM bicucullin and 2 μM strychnine.

Data were presented as mean ± SEM. Student's t-tests were used for statistical analysis and significance was considered at the level of the *p *< 0.05.

## Competing interests

The author(s) declare that they have no competing interests.
